# Parallels in the interactive effect of highly sensitive personality and social factors on behaviour problems in dogs and humans

**DOI:** 10.1038/s41598-020-62094-9

**Published:** 2020-03-24

**Authors:** Maya Bräm Dubé, Lucy Asher, Hanno Würbel, Stefanie Riemer, Luca Melotti

**Affiliations:** 1 Division of Animal Welfare, Vetsuisse Faculty, University of Berne, Berne, Switzerland; 2 Department for Small Animals, Clinic for Small Animal Surgery, Neurology, Vetsuisse Faculty, University of Zürich, Zürich, Switzerland; 3 Centre for Behaviour and Evolution, Institute of Neuroscience, Newcastle University, Newcastle, United Kingdom; 4 Department of Behavioural Biology, University of Münster, Münster, Germany

**Keywords:** Animal behaviour, Psychology

## Abstract

Sensory Processing Sensitivity (SPS) is a personality trait in humans characterised by a tendency to process information deeply, to be easily overstimulated, and to have strong emotional responses and an enhanced sensitivity to subtle stimuli. A trait similar to SPS has recently been identified in dogs (“canine Sensory Processing Sensitivity”, cSPS). In children, this trait interacts with parenting factors to influence emotional and mental development, which in turn are linked to behaviour problems. Paralleling these findings in humans, we demonstrate that cSPS interacts with owner personality and use of aversive communication to influence the likelihood of behaviour problems in dogs. More behaviour problems were reported for more highly sensitive dogs *per se*, when there was a relative mismatch between owner and dog personality, and when use of “negative punishment” was reported. These findings indicate that a dog’s personality might moderate how an individual is affected by environmental factors, particularly owner personality and communication style, emphasising the importance of considering individuality in prevention, development and treatment of behaviour problems in dogs.

## Introduction

Sensory Processing Sensitivity (SPS), also known as “high sensitivity”, is a personality trait in humans that involves a deeper than average processing of sensory information^1,2^, an enhanced perception of subtle stimuli^2–4^ and a strong emotional response to stimuli^3^. Recently, we identified a personality trait in the domestic dog which is comparable to SPS in humans, referred to as canine Sensory Processing Sensitivity (cSPS)^5^. The possibility to measure an analogous trait allows comparative research to be conducted and offers the potential to investigate interactive effects of dog and human personality.

Research has supported a number of parallels between dog-owner and child-parent relationships. The dog-owner bond, for example, fulfils the main characteristics of a human attachment bond^6–8^. In both species, behaviour problems and mental health problems are common and can significantly compromise psychological and emotional wellbeing^9,10^. There is some preliminary evidence to suggest that the same factors that influence the occurrence of behaviour problems in humans may also have this effect in dogs. Personality, for example has been shown to be linked with behavioural outcomes both in humans and dogs. For instance, in humans, SPS specifically is associated with a higher prevalence of certain mental health issues, such as the generalised subtype of anxiety disorder^11^, alexithymia, autism, depression, anxiety^12^), and avoidant and borderline personality disorders^13^. Dogs scoring higher in neuroticism are more likely to show separation-related behaviour problems^14^.

The extent to which predispositions to mental health or behaviour problems are expressed, however, depends not only on the personality of the affected individual, but also on environmental influences. Amongst these, parent personality, an important factor affecting parenting style, plays a crucial role in humans^15,16^. There is also evidence that owner personality influences a dog’s behaviour, including the occurrence of behaviour problems. Dogs owned by owners scoring high on neuroticism are more likely to suffer from separation-related problems^14^.

Another important environmental factor that affects behaviour is how information is conveyed from one individual to the other, which we refer to as “communication style”. Existing animal literature focusses primarily on the use of operant conditioning, and within this, mainly on the association of positive reinforcement and positive punishment with the occurrence of behaviour problems. There is data to date supporting a positive correlation between the use of aversive communication styles (primarily positive punishment, i.e. the addition of something aversive that leads to a decrease in the unwanted behaviour) and the occurrence of behaviour problems, both in humans^17–19^ and in dogs^20–25^. As positive punishment is perceived as “aversive”, it is thought to lead to high levels of arousal and negative emotions in the canine recipient^22,26^. Studies of the effect of removing or withholding something agreeable with the aim to decrease an unwanted behaviour (negative punishment) on behaviour are rare in the canine literature to date and are limited to case studies including few individuals (e.g.^27^) and interactions with personality have not been considered. As more highly sensitive individuals are more easily overstimulated and react with higher emotionality^3^, we might expect that they would be more strongly affected by aversive communication styles.

Moreover, the *interaction* of a child’s personality, including SPS, with parenting factors influences the occurrence of mental health and behaviour problems in humans. More highly sensitive individuals are more strongly affected by poor parenting quality than individuals scoring lower on the trait. In individuals scoring higher on sensitivity, this can manifest as an increased likelihood of shyness and negative affect^28^, a higher frequency of externalising behaviours^29^, and an increased risk of developing mental health issues, such as avoidant personality disorder^13^, anxiety and depression^30^. The effects of possible *interactions* of dog personality with owner factors to affect the dogs’ behaviour, however, have been rarely investigated. Working dogs scoring similar to their owners on analogous personality traits and sub-traits showed differences in performance, depending on the sub-traits that were matched. Dogs and owners matching on some sub-traits (human: ‘positive emotions’ and dog: ‘human familiarity’) increased performance while matching on others (human: ‘anxiety’, dog: ‘neuroticism’) decreased it^31^. Whilst a novel result, this study, however, did not use a validated method to assess canine personality, and some of the traits and sub-traits measured both in dogs and humans are not widely considered as personality traits.

Based on the existing knowledge of risk factors for behaviour problems in humans, the aim of this study was to investigate the individual and the interactive effects of dog cSPS personality, owner SPS personality and reported use of aversive communication style on the occurrence of behaviour problems in dogs. Using an online questionnaire, we tested the following hypotheses: (1) More behaviour problems in dogs are expected to be associated with higher cSPS (reflecting findings in humans where higher SPS is linked to a higher vulnerability to mental health issues); (2) More behaviour problems in dogs are expected when there is a mismatch between dog cSPS and owner SPS personalities, especially if the owners are less highly sensitive than the dogs; (3) More behaviour problems in dogs are expected to be associated with owner aversive communication style, with effects exaggerated in dogs scoring higher on cSPS.

## Methods

An international online questionnaire (published in^5^) served to test the hypotheses presented in this study. The questionnaire was available in English (Supplementary Table S1) and German (Supplementary Table S2) and consisted of three parts (see^5^ for details). Part 1 comprised general demographic information on the dogs and the owners, including presence of behaviour problems and questions regarding the use of different communication styles, i.e. techniques with the aim to reduce or increase specific behaviours by adding something aversive (referred to as “positive punishment” in this paper), withholding something agreeable (referred to as “negative punishment” in this paper), respectively, or adding something agreeable (referred to as “positive reinforcement” in this paper), as well as questions regarding factors that are described to influence behaviour, e.g. whether the dog had had a previous owner, degree of environmental stimulation, activity, and age at adoption. To evaluate behaviour problems, owners were asked whether their dog showed or had shown any behaviour they perceived as a problem, which they could reply to with yes or no. If yes was chosen, they were presented with a list of eight possible behaviour problem categories, from which they could select (see Supplementary Tables S1 and S2). Information from the online questionnaire did not allow the distinction between normal, but undesired behaviours (i.e. problems mainly based on training, education or environmental issues or those perceived as a nuisance by the owners) and pathological behaviour problems (i.e. behaviours which might correspond to mental health issues in humans). Therefore, in this study, any behaviour perceived as a problem by the owner is referred to as a “behaviour problem”. In order to assess communication style, owners were presented with the questions “How do you let your dog know when s/he does something right?” and “How do you let your dog know when s/he does something wrong?”. They were given a list of options, which were later used to categorise as to whether or not they used: “positive punishment” (i.e., addition of something aversive with the aim to reduce a behaviour), “negative punishment” (i.e., removal of something pleasant with the aim to reduce a behaviour), and “positive reinforcement” (i.e., addition of something pleasant with the aim to increase a behaviour). As this was a questionnaire-based survey without the possibility of direct observation, there was no means to determine how owners applied the techniques, e.g. regarding timing, consistency, intensity, etc. Information is, therefore, solely based on what the owners reported, hence we refer to the “reported” use. Use of “positive punishment” was assessed using 14 questions such as “I use my voice, e.g. shout or use a sharp tone of voice”, “I tug on the lead” or “I might kick or hit my dog”. Use of “negative punishment” was assessed using three questions “I withhold a reward (treats, petting, etc)”, “I give my dog a time-out, e.g. locking him/her away for a while” and “I ignore him/her”. Use of “positive reinforcement” was assessed using eight questions, such as I give him/her food treats”, “I pet or cuddle him/her” or “I praise him/her with my voice”) (see Supplementary Tables S1 and S2). Negative reinforcement (i.e. the removal of something aversive) was not considered, as owners are often unaware of this type of communication and it was, hence, not feasible to formulate unambiguous questions for the owners. Communication is a very complex process and its influence surpasses the simple knowledge of which communication style is implemented. For example, frequency and degree of application and especially timing are extremely important. Within the frame of this questionnaire-based study, it was, however, not possible to take these details into consideration.

Part 2 consisted of the validated “Highly Sensitive Dog Questionnaire”^5^, composed of 32 items scored by the owners on a 7-point Likert scale. The mean of all 32 items was used to calculate a “cSPS-score”, which ranged between 1 to 7 (the higher the score, the higher the dog’s sensitivity). Part 3 comprised the validated 27-item “Highly Sensitive Person Questionnaire”^32^, which was also scored on a 7-point Likert scale. The mean of all 27 Highly Sensitive Person questions was used to determine an “SPS-score”, ranging between 1 to 7 and representing the owner’s sensitivity.

### Statistical analysis

Statistical analysis was performed using R software version 3.02 (R Core Team, 2013). Generalised linear mixed effect models were used to investigate how the SPS-score, cSPS-score, and communication style interacted to influence the likelihood of behaviour problems in dogs. Alongside the personality and communication style variables, nine potentially confounding variables were included in the analysis, which led to a total of 13 potential predictors (see Table 1). To reduce the number of variables, screening tests and correlations between variables were performed as appropriate. Each potential confound was included as a fixed effect or covariate and as an interaction with cSPS in separate logistic regression models (glm with binomial link), with behaviour problems (yes or no) as the outcome variable in screening tests. Pearson’s and Spearman’s correlations were performed between potential confounds as appropriate. Potential confounding variables, which were associated with behaviour problems, were included in a final model selection if: (1) they were associated with behaviour problems (alone or as interaction with cSPS) at P < 0.1 and (2) they were not associated with other potential confounding variables. Forward stepwise selection (implemented using the R package lme 4.0 and the code glmer) was used to build a final model including predictors of screened confounding variables, in order of the lowest p-value from screening tests, with dog (cSPS) and owner (SPS) personality as covariates, reported use of “positive punishment” (Yes/No), “negative punishment” (Yes/No) and “positive reinforcement” (Yes/No) as fixed effects, and reported behaviour problems (Yes/No) as the outcome variable. Dog breed was included as a random effect. Interactions were tested for each confound included in the model and between the main variables of interest, dog and owner personality and communication style. Factors were retained in the model if they had a significant effect on model fit as tested using an ANOVA to compare models with and without each variable or interaction term. Model fit and model assumptions were checked and backwards elimination was used to ensure that the same variables would be retained in the final model. Due to the large sample size and potential for over-power, the effect sizes in this study were considered to be more informative than p-values. Odds ratios, Z values and p-values from the final model are presented.

**Table 1:** Independent variables tested for effects on the probability of behaviour problems in statistical models.

Dog and owner personality and communication style		
Predictor	Definition	Levels/Score
cSPS-score	Measurement of sensory processing sensitivity of the dog	Mean of 32 questions of the HSD questionnaire, continuous scale from 1–7, with 1 being low and 7 high
SPS-score	Measurement of sensory processing sensitivity of the owner	Mean of 27 questions of the HSP questionnaire, continuous scale from 1–7, with 1 being low and 7 high
Reported “negative punishment”	Use of techniques with intention to reduce behaviour by withdrawing something agreeable	Binomial yes or no
Reported “positive punishment”	Use of techniques with the intention to reduce behaviour by adding something aversive	Binomial yes or no
**Potential confounding variables**		
**Predictor**	**Definition**	**Levels/Score**
Stimulation first environment	Degree of stimulation in first few months of life	5-point Likert scale: 1 very little, 5 a lot
Stimulation current environment	Degree of stimulation in current living situation	5-point Likert scale: 1 very little, 5 a lot
Previous owner	Whether the dog had a previous owner or not	Categorical with 3 options: No, Yes; ‘I don’t know’
Dog sex	Sex and neuter status of the dog	Categorical with 4 options: Male intact, male neutered, female intact, female neutered
Active time	Active time spent with the dog: walking, playing, working	Categorical with 4 options: <1 hour, 1–3 hours,> 3 hours, ‘I don’t know’ (coded as missing data)
Adoption Age	Age at which the dog was acquired	Continuous: Age in months
Dog Age	Current age of the dog	Continuous: Age in years
Number of people in household	Number of people currently living in the same household as the dog	Categorical with 3 options: only me, two people, more than two people
Dog weight		Continuous in kg

SPS = Sensory Processing Sensitivity, cSPS = canine Sensory Processing Sensitivity.

## Results

### Descriptive results

The questionnaire was completed for 3647 dogs, 50% of which were male (68% neutered) and 50% female (76% neutered). The mean weight of the dogs was 21.3 (SD: ± 11.6) kg, the mean age 5.8 (±3.6) years and the mean age at adoption was 11 (±2.9) months. More detailed information regarding demographics is provided in our previous publication^5^.

The owners of 42% of the dogs stated that their dog had a behaviour problem. All owners but three reported using “positive reinforcement” methods, hence this factor was not considered in further analyses. Use of “positive punishment” methods was reported by 73% of owners and use of “negative punishment” by 67%. The cSPS-score ranged from 1.4 to 6.7 with a mean of 4.0 (±0.9) and the SPS-score ranged from 1.4 to 7 with a mean of 4.2 (±1.1). The cSPS-score was normally distributed, whereas the SPS-score was left-skewed (skewness = 0.44), which suggests that the tail/spike in the area of high scores was longer.

The mean for environmental stimulation was 3.1 (±1.3) on a Likert Scale of 1–5 in the first months of life and 2.95 (±1.2) in the current living environment. A total of 26% of dogs had had a previous owner, 65% did not; the rest of owners did not know or did not reply to the question.

The owners of 14% of the dogs offered less than an hour of activity per day, 70% between 1–3 hours and 16% more than three hours. In 49% of the households there were two people, in 33% there were more than two and in 18% only one person.

### Hypothesis 1: Behaviour problems are more likely in dogs with higher cSPS-scores

**Figure 1:**
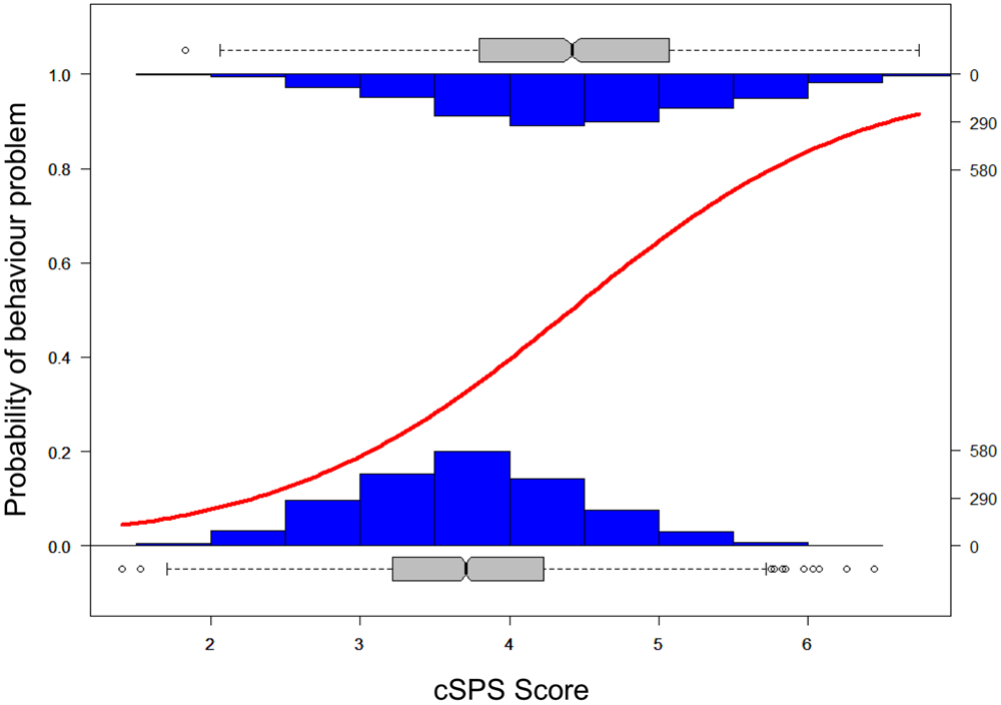
The probability of behaviour problems increased with higher cSPS-score based on fitted logistic regression curve. Histogram of c-SPS scores and box plots (with median and interquartile range, upper and lower extremes and outliers as dots) shown for participants who reported their dog did (1) and did not (0) have a behaviour problem.

As predicted, cSPS-scores were associated with the occurrence of behaviour problems: owners of more highly sensitive dogs were more likely to report behaviour problems than those of less highly sensitive dogs (see Table 2 and Fig. 1). This was the most predictive effect in the statistical model of the probability of behaviour problems, compared to other independent variables and confounds.

**Table 2:** Results of the final model of the effect of canine Sensory Processing Sensitivity (cSPS-score), owner Sensory Processing Sensitivity (SPS-score) and aversive communication style (reported “negative” and “positive punishment”) on the likelihood of behaviour problems.

Predictor	Odds ratio (95% CI)	Z	P value
cSPS-score	3.86 (3.48–4.28)	6.62	<0.0001
SPS-score	1.47 (1.24–1.76)	2.22	0.0262
cSPS-score x SPS-score	0.89 (0.86–0.93)	−2.56	0.0104
Reported “positive punishment”	1.27 (1.17–1.38)	2.78	0.0055
Reported “negative punishment”	0.55 (0.36–0.83)	−1.45	0.1462
cSPS-score x reported “negative punishment”	1.24 (1.13–1.37)	2.21	0.0269
Dog sex: male neutered compared to male intact†	1.33 (1.19–1.49)	2.46	0.0141
Previous owner: No previous owner compared to previous owner‡	0.74 (0.68–0.81)	−3.33	0.0009

^†^Female intact and neutered were not significantly different to male intact. ^‡^Previous owner = “don’t know” was not significantly different from no previous owner.

### Hypothesis 2: Behaviour problems are more likely with a mismatch between cSPS and SPS

**Figure 2:**
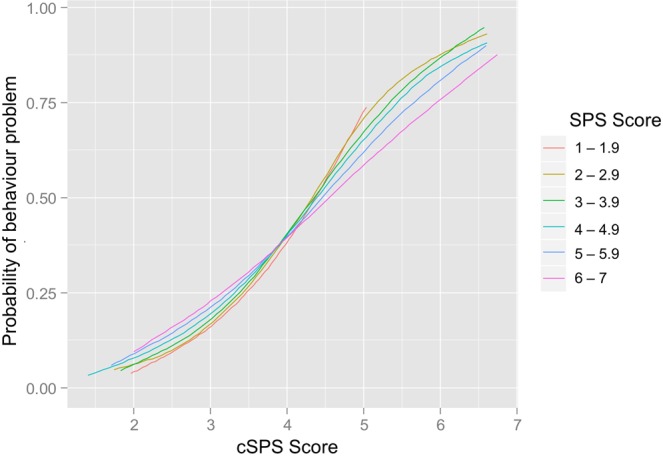
Interaction effect between canine Sensory Processing Sensitivity (cSPS)-score and Sensory Processing Sensitivity (SPS)-score on the occurrence of behaviour problems. Fitted logistic curves are shown for each unit increase in SPS score.

Independent of dog personality, owners who scored higher on SPS were more likely to report their dogs as having behaviour problems in the questionnaire (see Table 2). As predicted, the occurrence of behaviour problems was affected by an interaction between dog and owner personality, whereby a relative personality mismatch between dog and owner was associated with more behaviour problems (Fig. 2, Table 2). This effect was particularly strong if the dog scored higher than its owner on the sensitivity scale. For each increase in the cSPS-score of the dog, the odds of having a behaviour problem increased by 3.86, but for dogs with higher cSPS-scores, their owners’ sensitivity score being more dissimilar to their own increased these odds by 1.11 (Table 2).

### Hypothesis 3: Behaviour problems are more frequent in dogs scoring higher on cSPS when aversive communication styles are used

**Figure 3:**
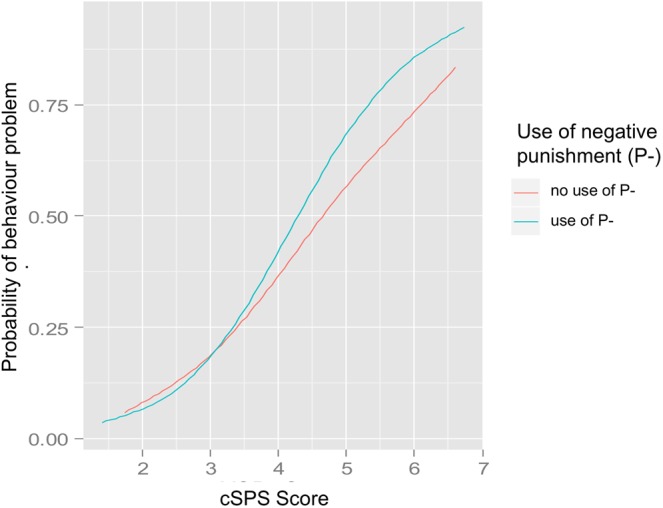
The effect of the interaction of canine Sensory Processing Sensitivity-score (cSPS-score) and reported “negative punishment” (P-) on the outcome of behaviour problems. Fitted logistic curves are shown for participants that reported using and not using “negative punishment”.

While reported “positive punishment” was associated with behaviour problems (the odds of having a behaviour problem were 1.27 times higher if owners reported the use of “positive punishment”), contrary to our hypothesis, there was no interaction with cSPS. There was no main effect of the reported use of “negative punishment” on the probability of behaviour problems. However, there was a statistically significant interaction between cSPS-score and reported use of “negative punishment” on the outcome of behaviour problems: the odds of showing a behaviour problem increased by 1.24 if “negative punishment” was reported, but only in dogs with higher cSPS-scores and not those with lower scores (Fig. 3, Table 2).

### Associations of confounding variables with the occurrence of behaviour problems

Other variables which were found to be associated with behaviour problems were dog sex and previous owner. Neutered males were more likely to have behaviour problems reported than intact males (Table 2). Neutered and intact females did not differ in the occurrence of behaviour problems from the comparator group of intact males. Dogs who had a previous owner were also more likely to have behaviour problems (Table 2).

## Discussion

Using validated questionnaires to assess the SPS trait in humans and the cSPS trait in dogs, this study demonstrates that personalities not only interact within a species, but potentially also across species between dog and human, to affect the likelihood of behaviour problems. The results from this study indicate that similar personality and environmental factors may underlie behaviour problems in pet dogs and humans. In parallel with findings in humans, of all the factors included in this study, cSPS had the highest association with the occurrence of behaviour problems, suggesting a link between dog personality and behaviour problems. Congruent with findings in parent-child studies, an interactive effect of dog personality with owner personality and aversive communication style on the occurrence of behaviour problems was found. Dogs showed more behaviour problems when there was a relative mismatch between owner and dog sensitivity, especially if the dog scored higher on this trait than the owner. Moreover, there were interactive effects of cSPS in dogs and reported “negative punishment” on the occurrence of behaviour problems: Dogs scoring higher on cSPS, but not those scoring lower, showed an increased incidence of behaviour problems when “negative punishment” was reported. Confirming previous findings in dogs and humans, reported “positive punishment” was associated with a greater probability of behaviour problems. This association, however, was independent of personality.

There are several possible explanations for the relationship between high sensitivity (SPS/cSPS) and mental health and/or behaviour problems in both humans and dogs. Firstly, mental health and behaviour problems in humans^33,34^ as well as behaviour problems in animals^35–37^ are influenced by the ability of the individual to cope with stress. What is perceived as stressful depends on genetic predisposition, experience throughout life, individual differences in appraisal of the stimulus^38,39^ and epigenetic influences^40^. One characteristic of SPS in humans is a greater sensory sensitivity to details, involving more subtle registering and deeper processing of these^1,3,4^. This is linked to being more easily overwhelmed by sensory input, which in turn is associated with the initiation of the physiological stress response. Indeed, humans scoring higher on SPS reported higher perceived stress levels than those scoring lower on the trait^41,42^, making them more vulnerable to the effects of stress. If cSPS in dogs really is analogous to SPS in humans, dogs scoring higher on the trait might be expected to have an increased susceptibility to stress and/or a lower threshold for stress to be induced, and therefore also an increased likelihood to develop behaviour problems. The results of this study provide first indications that this might be the case.

A second and related potential explanation for the relationship between cSPS and behaviour problems is that SPS has been proposed to be linked with differential environmental sensitivity^43^. Environmental sensitivity is defined as “the degree to which an individual may register, process and respond to external factors”^44^ [p.1] and includes greater responsiveness to both positive and negative stimuli^43,45^. In humans, the study of such variability of sensitivity to environmental factors has particularly focused on the interaction of child personality with parenting style^46–48^, which in turn is influenced by parent personality^49^. Children scoring higher on SPS react more negatively to verbal and psychological hostility, psychological control and coercive methods, which can be considered aversive styles of communication^43,50,51^. However, children scoring high on SPS also react more to positive (supportive, warm and responsive, autonomy granting, use of positive reinforcement) and inductive discipline parenting effects^43,48^. They show more externalising behaviour with negative parenting and less externalising behaviour with more positive parenting than children with average or lower SPS scores^29^. Similar to more highly sensitive children, the results of the present study suggest that dogs scoring higher on cSPS are more strongly affected by owner factors (in our case personality and reported “negative punishment”) than those scoring lower. It is possible that differential environmental susceptibility, including the higher responsiveness to positive experiences, also applies to dogs; however, we do not have the data to address the latter in the current study. To investigate this further we suggest prospective, longitudinal studies including animals exposed to different types of environments and communication styles (e.g. different types of puppy classes and training approaches) or investigation of the association of cSPS with the selection for and success in working dogs, with the inclusion of data collected on positive, as well as negative, experiences.

A mismatch of personalities of owner and dog was associated with a higher reporting of behaviour problems in dogs. One possible explanation for this is that different personalities have different needs. The needs of another individual are most easily recognised and fulfilled if an individual can empathise with the other. This is true within the human species, e.g. children experiencing more child-directed empathy by their parents show less conduct problems^52^. As noted above, the human-dog bond can to a certain extent be compared to that between a parent and child^53,54^. Based on this and the similarities found in our studies regarding SPS and cSPS in humans and dogs, it could be suggested that the closer in personality owners and dogs are, the more likely the owner can empathise with the dog, and the greater the understanding of the dog’s needs. Owners with similar needs to their dogs’ might recognise signs of fear and stress earlier and adjust the situation accordingly or avoid it, whereas those different in personality might not be aware of a problem. Owners closer in personality are also likely to choose a lifestyle that inadvertently better suits their pet’s needs.

The information conveyed by the owner to the dog depends on choice of communication style, simplified in this study by using the four quadrants of operant conditioning. These consist of positive and negative reinforcement and positive and negative punishment. Both positive and negative punishment are defined as decreasing the likelihood of an undesired behaviour, the former by adding something disagreeable, the latter by withdrawing something agreeable^25^. The use of both types of punishment is associated with negative affect^55^ and is, hence, considered to be aversive. The current study only assessed correlations, and hence does not allow to distinguish whether more aversive communication (reported “positive and negative punishment”) was a predictor or consequence of behaviour problems. It is possible that owners of dogs with behaviour problems use more aversive communication with the aim to decrease the occurrence of the undesired behaviour. However, existing literature does suggest that the use of aversive communication styles (primarily “positive punishment”) can influence behaviour problems both in humans^18^ and dogs^20,22,23,25,26^. Our hypothesis was that dogs higher on cSPS would be more strongly affected by aversive communication and, therefore, show more behaviour problems than less highly sensitive individuals. However, this was only partially confirmed: A higher incidence of behaviour problems was observed with the reported use of “positive punishment”, but independent of cSPS. A personality effect was, however, found for reported “negative punishment”. The link between negative punishment and behaviour problems has been largely neglected in the canine literature to date. As cSPS is associated with a higher incidence of behaviour problems, it could be suggested that owners of more highly sensitive dogs are also more likely to report the use of any punishment style, including negative punishment. Another possible explanation is that more highly sensitive dogs are differentially affected by the use of negative punishment. In humans, severe and/or repetitive withdrawal of something desired (e.g. ignoring, rejecting, isolating, etc.) has been described to be associated with adverse consequences for the wellbeing of children^56–58^. It will be important in future research to not just consider associations between canine welfare/behaviour problems and positive punishment, but to include negative punishment as well. Further, more research is warranted into the potential differential influences of aversive communication styles and personality traits such as cSPS.

In summary, the results of this study provide evidence for parallel social influences on behaviour problems in dogs and humans, which occur not only within each species, but also across human-dog relationships. The online nature of this study somewhat limits the possibilities of interpretation. For one, it was not possible to differentiate between normal, but disturbing behaviours and actual pathological behaviour problems (which might parallel mental health problems in humans). Further analyses of the different subtypes of behaviour problems might provide more context with which to interpret these results. Although we find parallels between human-human and human-dog relationships, it is possible that the underlying mechanisms are not shared. Secondly, the interpretation of communication styles was based on the owners’ reports, which did not allow to evaluate important aspects such as timing, frequency and intensity of communication styles. Addressing this was beyond the scope of this study and would necessitate observations in experimental settings. However, these aspects are important for a complete understanding of the human-dog communication and how this could potentially affect the emotional and arousal states, as well as the behaviours of both individuals. Thirdly, despite by-proxy questionnaires being widely used both in studies involving human infants (e.g.^46^ as well as animals^59,60^, there remains a certain “effect of owner”, which cannot be eliminated. And lastly, the set-up of this study does not allow conclusions regarding causality, but only regarding associations. Longitudinal, prospective studies involving direct observation of human-dog interactions would be necessary to specifically study and draw clear conclusions about causality.

Nevertheless, this is the first study to assess the interactive effects of the same personality trait in two different species on behaviour problems, using validated personality measures in both species. The parallel findings between humans and dogs with regard to SPS broaden the options for future research. Firstly, future studies could focus upon the interaction of personality with environmental – particularly social cross-species – factors and associations with welfare and behaviour. Secondly, highly sensitive dogs may be considered as a possible model species for studying highly sensitivity humans. On the practical side, this knowledge particularly emphasises the importance of considering the individual and its susceptibility to environmental influences, including communication style, in the prevention, development and the treatment of behaviour problems. Matching dog and owner personalities could be used to inform future rehoming strategies. Dogs could be matched to potential owners by personality, particularly for highly sensitive dogs, or training could be provided to help owners to better recognise the needs of dogs with high sensitivity. Similarly, if findings hold in future studies, then owner education may be warranted into the differential susceptibility of individuals to personality and communication style.

## Supplementary Information

Supplementary Information.

Supplementary Information2.
