# Supplementary material for: Parallels in the interactive effect of highly sensitive personality and social factors on behaviour problems in dogs and humans
Source: Sci Rep. 2020 Mar 24;10:5288. doi: 10.1038/s41598-020-62094-9 (PMC7093480; doi:10.1038/s41598-020-62094-9)
Supplement: Supplementary file 1 — Supplementary Information. [file 41598_2020_62094_MOESM1_ESM.pdf]

Parallels in the interactive effect of highly sensitive personality and social factors on behaviour problems in dogs and humans

Maya Bräm Dubé, Lucy Asher, Hanno Würbel, Stefanie Riemer, Luca Melotti

Questionnaire in English

Question / **Reply options**

Owner information

Question	Reply options
Would you like feedback on the results of the study? If yes, please give us your email address in the textbox below	Yes/no<br>short free text
In which country do you currently live?	**Dropdown menu**<br>· Austria<br>· Canada<br>· Germany<br>· Switzerland<br>· United Kingdom<br>· United States of America<br>· Other Country
What is your age?	**categories:**<br>< 18 years<br>18-30 years<br>31-65 years<br>> 65 years
What is your gender?	· male<br>· female
Do you belong to any of the following professional / academic categories?	**Multiple Choice List**<br>· dog trainer<br>· dog trainer specialised in behaviour<br>· veterinarian<br>· behaviour veterinarian<br>· university student<br>· university employee<br>· none of the above
How did you hear about this study?	· Flyer<br>· Email<br>· Other (with comment option)

Dog information

Question	Reply options
What is your dog's name?	short free text
How old is your dog at this point in time? (if less than a year, please indicate 0 in the year box)	years and months
How old was your dog when you got him/her?<br>If less than a year, please indicate 0 in the year box. If you don't know exactly, please provide your best estimate.	years and months
What breed is your dog? If s/he is a mixed breed, please indicate "mixed breed"	short free text
What is your dog's gender?	**Categories**<br>· male intact<br>· male neutered<br>· male intact<br>· male neutered
What is your dog's current weight?<br>Please give us your dog's weight in numbers only and indicate whether it is in lb or kg.	kg or lb<br>short free text

Communication / Training

Question	Reply options
How do you let your dog know when s/he does something right?<br>*(comment: all techniques in the reward category were considered to be positive reinforcement (R+), but not indicated as such in the questionnaire available to participants)*	**Multiple Choice List**<br>· I give him/her food treats (R+)<br>· I pet and/or caress him/her (R+)<br>· I use a clicker or marker word (R+)<br>· I praise him/her with my voice (R+)<br>· I play with him/her and/or give him/her a toy (R+)<br>· I work with a prey dummy (R+)<br>· I give him/her friendly attention (R+)<br>· I reward my dog with other things s/he enjoys, e.g. take her for a walk, let him play, let her sniff, let him dig, etc) (R+)<br>· other<br>· none of the above
How do you let your dog know when s/he does something wrong?<br>(*comment: retrospective categories given in parentheses after the responses: positive punishment (P+), negative punishment (P-), this information was not available to the participants*)	**Multiple Choice List**<br>· I withhold a reward (treats, petting, etc.) (P-)<br>· I use my voice, e.g. shout or use a sharp tone of voice (P+)<br>· I turn him/her onto his/her back (P+)<br>· I give him/her a time-out, e.g. by locking him/her away for a while (P-)<br>· I press my dog to the ground (P+)<br>· I do obedience work (P+)<br>· I use a spray collar (P+)<br>· I ignore him/her (P-)<br>· I use a shock collar (P+)<br>· I make him/her submit (P+)<br>· I tug on the lead (P+)<br>· I put my hand over his/her muzzle (P+)<br>· I use a choke collar (P+)<br>· I use a noise like keys/discs/a bottle or can filled with stones or similar to stop the behavior (P+)<br>· I might kick or hit my dog (P+)<br>· I spray my dog with water (P+)<br>· I tap my dog on the nose (P+)<br>· other<br>· none of the above

Health

Does your dog suffer or has s/he in the past suffered from any physical illnesses? If yes, please briefly describe	yes/no<br>-> if yes, please indicate what category of physical illness it is/was.<br><br>· Digestive tract (e.g. diarrhea, constipation, vomiting, tooth problems, salivation, food intolerance, etc.)<br>· Respiratory tract (e.g. coughing, sneezing, secretion out of nostrils, sounds when breathing, etc.)<br>· Cardiovascular system (e.g. heart murmur, out of breath quickly, irregular heart beat, etc.)<br>· Skin (e.g. infections, itchiness/scratching, hair loss, ear infections, wounds, etc.)<br>· Sensory organs (e.g. decreased vision, cataract, hearing loss, etc.)<br>· Urinary tract (e.g. bladder infections, kidney problems, etc.)<br>· Reproductive tract (e.g. cryptorchism, pyometra, prostate problems, etc.)<br>· Immune system (e.g. allergies, auto-immune disease, reaction to vaccinations, frequent inflammations, etc.)<br>· Locomotor apparatus (e.g. arthritis, fractures, limping, gait abnormalities, muscle atrophy, trembling, etc.)<br>· Nervous system (e.g. seizures/epilepsy, cauda equina, etc.)<br>· Metabolic (e.g. diabetes mellitus, hypothyroidism, Cushings, Addisons, etc.)<br>· Infections (e.g. bacterial, viral, fungal, tick-borne disease (e.g. borreliosis), leishmaniosis, etc.)<br>· Surgeries (e.g. castration / sterilisation, fractures, gastric volvulus, (bite-) wounds, etc.)<br>· Other
Does your dog show or has s/he shown any behavior problems or behaviours that bother you?	yes/no<br>-> if yes, please indicate what type of behaviour problems these are/were.<br><br>· Aggression (e.g. towards people, towards other dogs, when touched)<br>· Fears / Phobias / Anxiety (e.g. afraid of fireworks or thunderstorms, always afraid and tense while outside, afraid of people, afraid of other dogs, crowded places)<br>· Separation-related problems (e.g. vocalisation, destruction, house soiling when alone or separate from you)<br>· Excessive behaviour (e.g. hyperactivity, hyperreactivity, hypervigilance, cannot stop)<br>· Diminished activity (e.g. depression, lack of energy)<br>· Elimination problems (e.g. house soiling with urine and/or faeces)<br>· Repetitive behaviour, stereotypic and/or compulsive behaviours (e.g. tail chasing, licking itself, turning in circles, chasing shadows)<br>· Travel-related problems (e.g. cannot relax in the car, barks, shows signs of car sickness)<br>· other

Surroundings

How many people live in the same household as your dog (including yourself)?	· only me<br>· 2 people<br>· more than 2 people
Please quantify the degree of stimulation (e.g. noise, amount of traffic, people, dogs, other animals, etc.) in your dog's living surroundings.<br>· for current living surroundings<br>· for first living surroundings	Likert scale from 1 - 5, with 1 = very quiet and 5 = very loud<br>I don't know

Dog's history

What country did you get your dog from?	· from the same country in which s/he lives now<br>· from abroad
Did your dog have any previous owner/s (apart from the breeder)?	· yes<br>· no<br>· I don't know

Personality / Activity

Do you consider your dog to be any of the following? If yes, why?	answer options: yes / no / I don't know<br><br>· fearful<br>· neurotic<br>· curious<br>· sensitive<br>· shy<br>· uncertain<br>· none of the above
How much "active" time does your dog receive per day (walks, playing in the yard / inside, training, etc.)	< 1 hour<br>1-3 hours<br>> 3 hours<br>I don't know
When confronted with a new, unfamiliar object, how is your dog most likely to react?	· S/he stops and watches from a distance<br>· S/he starts barking<br>· S/he moves away and avoids it<br>· S/he moves towards it and starts playing<br>· None of the above<br>· I don't know

Question number	**The Highly Sensitive Dog Questionnaire**<br>Likert scale 1-7, with 1 = not true at all; 4 = more or less true; 7 = completely true<br>includes the option "I don't know"
1	My dog is easily stressed, is easily overwhelmed by situations.
2	My dog notices small changes.
3	My dog startles easily.
4	My dog gets nervous quickly or is often nervous.
5	My dog seems to absorb everything that is happening around him.
6	My dog reacts when we argue at home.
7	My dog tends to be uncertain and/or careful.
8	My dog is emotionally stable, i.e. he is mostly even-tempered and not easily unnerved.
9	My dog has a tendency to be mistrustful.
10	My dog tends to be restless.
11	My dog easily adapts to a new environment and can relax there.
12	My dog has a subtle perception, i.e. he notices a lot or almost everything.
13	My dog has problems adapting to changes in every day life (e.g. changes in routine, visitors, etc.) and/or bigger changes in life (e.g. change of partner, rearranging furniture, going on holidays, moving home, etc.).
14	My dog reacts to small changes in voice, i.e. changes in intonation and volume.
15	It takes a long time for my dog to calm down after an arousing event.
16	My dog has trouble when people touch him and/or when things touch him (e.g. harness, coat, wet leaves, etc.)
17	My dog is reactive, i.e. he quickly perceives small stimuli and reacts quickly and/or strongly to them.
18	My dog is attentive.
19	My dog seems thoughtful.
20	My dog observes everything that is happening around him.
21	My dog always "has his antennae up".
22	My dog is generally relaxed, can cope well with stress.
23	My dog has problems when he is left alone outside and I move out of sight.
24	My dog reacts strongly to punishment.
25	My dog is biddable.
26	My dog is sensitive.
27	My dog is demanding.
28	My dog is emotional, i.e. reacts strongly to positive and/or negative events.
29	My dog needs a sense of security.
30	My dog is easily excitable, be it through positive or negative stimuli.
31	My dog is intelligent.
32	My dog reacts strongly to visual stimuli.

(highlighted) questions that need to be reversed for analysis, i.e. 1=7, 2=6, 3=5, 4=4

Question number	**The Highly Sensitive Person Questionnaire**<br>Likert Scale from 1-7 with 1 = not true at all; 4 = more or less true; 7 = completely true
1	Are you easily overwhelmed by strong sensory input?
2	Do you seem to be aware of subtleties in your environment?
3	Do other people's moods affect you?
4	Do you tend to be more sensitive to pain?
5	Do you find yourself needing to withdraw during busy days into bed or into a darkened room or any place where you can have some privacy and relief from stimulation?
6	Are you particularly sensitive to the effects of caffeine?
7	Are you easily overwhelmed by things like bright lights, strong smells, coarse fabrics, or sirens close by?
8	Do you have a rich, complex inner life?
9	Are you made uncomfortable by loud noises?
10	Are you deeply moved by the arts or music?
11	Does your nervous system sometimes feel so frazzled that you just have to go off by yourself?
12	Are you conscientious?
13	Do you startle easily?
14	Do you get rattled when you have a lot to do in a short amount of time?
15	When people are uncomfortable in a physical environment, do you tend to know what needs to be done to make it more comfortable (like changing the lighting or the seating)?
16	Are you annoyed when people try to get you to do too many things at once?
17	Do you try hard to avoid making mistakes or forgetting things?
18	Do you make a point to avoid violent movies and TV shows?
19	Do you become unpleasantly aroused when a lot is going on around you?
20	Does being very hungry create a strong reaction in you, disrupting your concentration or mood?
21	Do changes in your life shake you up?
22	Do you notice and enjoy delicate or fine scents, tastes, sounds, works of art?
23	Do you find it upleasant to have a lot going on at once?
24	Do you make it a high priority to arrange your life to avoid upsetting or overwhelming situations?
25	Are you bothered by intense stimuli, like loud noises or chaotic scenes?
26	When you must compete or be observed while performing a task, do you become so nervous or shaky that you do much worse than you would otherwise?
27	When you were a child, did parents or teachers seem to see you as sensitive or shy?
