# Supplementary material for: Parallels in the interactive effect of highly sensitive personality and social factors on behaviour problems in dogs and humans
Source: Sci Rep. 2020 Mar 24;10:5288. doi: 10.1038/s41598-020-62094-9 (PMC7093480; doi:10.1038/s41598-020-62094-9)
Supplement: Supplementary file 2 — Supplementary Information2. [file 41598_2020_62094_MOESM2_ESM.pdf]

Parallels in the interactive effect of highly sensitive personality and social factors on behaviour problems in dogs and humans

Maya Bräm Dubé, Lucy Asher, Hanno Würbel, Stefanie Riemer, Luca Melotti

Fragebogen Deutsch

Frage	Antwortoptionen
**Besitzer**	
Möchten Sie über die Resultate dieser Studie informiert werden? Falls ja, bitte geben Sie uns im Kommentarfeld Ihre Emailadresse an.	Ja/nein<br>kurzer Freitext
In welchem Land wohnen Sie momentan?	**Drop-down Liste**<br>· Österreich<br>· Kanada<br>· Deutschland<br>· Schweiz<br>· Vereinigtes Königreich<br>· Vereinigte Staaten von Amerika<br>· Anderes Land
Wie alt sind Sie?	**Kategorien**<br>< 18 Jahre<br>18-30 Jahre<br>31-65 Jahre<br>> 65 Jahre
Welches ist Ihr Geschlecht?	· männlich<br>· weiblich
Gehören Sie einer der folgenden beruflichen oder akademischen Kategorien an ?	**Multiple Choice**<br>· Hundetrainer<br>· Verhaltenstherapeut/in für Hunde<br>· Tierarzt/Tierärztin<br>· Verhaltensmediziner/in<br>· Student/in<br>· Universitätsangestellte/r<br>· Keines davon trifft zu
Wie haben Sie von dieser Studie erfahren ?	· Flyer<br>· Email<br>· Sonstiges (mit Kommentar-Option)

Informationen zum Hund

Was ist der Name Ihres Hundes?	Kurzer Freitext
Wie alt ist Ihr Hund jetzt?	Jahre und Monate
Wie alt war Ihr Hund, als Sie ihn übernommen haben?	Jahre und Monate
Welche Rasse ist Ihr Hund? Falls er/sie ein Mischling ist, bitte wählen Sie die Option "Mischling".	Kurzer Freitext
Was ist das Geschlecht Ihres Hundes?	**Kategorien**<br>· männlich unkastriert<br>· männlich kastriert<br>· weiblich unkastriert<br>· weiblich kastriert
Was ist das jetzige Gewicht Ihres Hundes?<br>Bitte geben Sie das Gewicht Ihres Hundes nur in Zahlen an und geben sie an, ob es sich um lb oder kg handelt.	kg oder lb<br>kurzer Freitext

Kommunikation / Training

Wie lassen Sie es Ihren Hund wissen, wenn er/sie etwas richtig macht ?<br>*(Kommentar: Alle Techniken innerhalb dieser Kategorie wurden als positive Verstärkung interpretiert (R+). Diese Information stand den Teilnehmern nicht zur Verfügung)*	**Multiple Choice Liste**<br>· Ich gebe ihm/ihr Leckerlis (R+)<br>· Ich streichle ihn/sie (R+)<br>· Ich arbeite mit einem Klicker oder mit einem Marker Wort (R+)<br>· Ich lobe ihn/sie mit meiner Stimme (R+)<br>· Ich spiele mit ihm/ihr oder gebe ihm/ihr ein Spielzeug (R+)<br>· Ich arbeite mit einem Prey-Dummy (R+)<br>· Ich gebe ihm/ihr meine freundliche Aufmerksamkeit (R+)<br>· Ich belohne meinen Hund mit anderen Dingen, die sie/er gerne hat (z.B. gehe mit ihm spazieren, lasse ihn spielen, lasse ihn schnüffeln, erlaube ihm zu graben, etc.) (R+)<br>· Anderes<br>· Nichts vom oben genannten trifft zu
Wie lassen Sie es Ihren Hund wissen, wenn er/sie etwas falsch macht ?<br>*(Kommentar: Retrospektive Kategorisierung ist in Klammern angegeben: Positive Bestrafung (P+), negative Bestrafung (P-). Den Teilnehmern stand diese Information nicht zur Verfügung)*	**Multiple Choice Liste**<br>· Ich gebe ihm/ihr bewusst die erwartete Belohnung (z.B. Futter, Streicheleinheiten) nicht (P-)<br>· Ich setze meine Stimme ein, schreie ihn/sie z.B. an oder verwende einen harten Ton (P+)<br>· Ich drehe ihn/sie auf den Rücken (P+)<br>· Ich gebe meinem Hund ein Timeout, indem ich ihn z.B. in ein anderes Zimmer sperre oder ihn/sie in seine/ihre Box schicke (P-)<br>· Ich drücke meinen Hund zu Boden (P+)<br>· Ich mache ein paar Obedience Übungen (P+)<br>· Ich verwende ein Sprühhalsband (P+)<br>· Ich ignoriere ihn/sie (P-)<br>· Ich verwende ein Elektroschockhalsband (P+)<br>· Ich lasse ihn/sie sich unterordnen (P+)<br>· Ich gebe ihm/ihr einen Leinenruck (P+)<br>· Ich verwende den Schnauzengriff (P+)<br>· Ich verwende ein Zughalsband (P+)<br>· Ich verwende ein Geräusch, z.B. Wurfscheiben / eine mit Steinen gefüllte Flasche oder Büchse (oder ähnliches), um das Verhalten zu beenden (P+)<br>· Es kann vorkommen, dass ich meinen Hund schlage oder einen Tritt gebe (P+)<br>· Ich spritze meinen Hund mit Wasser an (P+)<br>· Ich gebe ihm/ihr eine kleinen Klaps auf die Nase (P+)<br>· Anderes<br>· Nichts vom oben genannten trifft zu

Gesundheit

Leidet Ihr Hund an einer schlimmen, langandauernden oder immer wiederkehrenden körperlichen Erkrankung, oder hat er/sie je unter einer solchen gelitten ?	Ja/nein<br>-> falls ja, geben Sie bitte an, um welche Art der körperlichen Erkrankung es sich handelt/e.<br><br>· Verdauungstrakt (z.B. Durchfall, Verstopfung, Erbrechen, Zahnprobleme, Speicheln, Futterunverträglichkeit, etc.)<br>· Atemwege (z.B. Husten, Niesen, Nasenausfluss, Atemgeräusche, etc.)<br>· Herz-Kreislauf-System (z.B. Herzgeräusch, unregelmässiger Herzschlag, schnell ausser Atem, etc.)<br>· Haut (z.B. Infektionen, Juckreiz/Kratzen, Haarverlust, Ohrinfektionen, Wunden, etc.)<br>· Sinnesorgane (z.B. vermindertes Sehvermögen/Blindheit, grauer Star, Taubheit, etc.)<br>· Harntrakt (z.B. Blasenentzündung, Nierenprobleme, etc.)<br>· Fortpflanzungsorgane (z.B. Gebärmutterentzündung, Prostataprobleme, Kryptorchismus, etc.)<br>· Immunsystem (z.B. Allergien, Autoimmunkrankheiten, Impfreaktionen, häufige Entzündungen, etc.)<br>· Bewegungsapparat (z.B. Arthrose, Knochenbrüche, Hinken, seltsamer Gang, Muskelschwund, Zittern, etc.)<br>· Nervensystem (z.B. Epilepsie, Cauda equina, etc.)<br>· Stoffwechsel (z.B. Diabetes mellitus, Schilddrüsenunterfunktion, Cushing, Addison, etc.)<br>· Infektionen (z.B. bakterielle oder virale Infektionen, Pilzbefall, Zecken-assoziierte Erkrankungen (z.B. Borreliose), Leishmaniose, etc.)<br>· Operationen (z.B. Kastration/Sterilisation, Knochenbrüche, Magendrehung, Bisswunden, etc.)<br>· Anderes
Zeigt Ihr Hund irgendwelche Verhaltensprobleme oder Verhaltensweisen, die Sie stören ?	Ja/nein<br>-> falls ja, geben Sie bitte an, welche Art von Verhaltensproblemen dies sind/waren.<br><br>· Aggression (z.B. gegen Leute, andere Hunde, bei Berührung, etc.)<br>· Angst / Phobien / Furcht (z.B. Angst vor Geräuschen wie Feuerwerk oder Gewitter, Anspannung und Ängstlichkeit wenn ausser Haus, Angst vor Personen, Angst vor anderen Hunden, Angst vor Menschenmengen)<br>· Probleme alleine zu bleiben (z.B. Vokalisation, Zerstörung, Unsauberkeit, wenn alleine zuhause oder von Ihnen getrennt)<br>· Exzessives Verhalten (z.B. Hyperaktivität, Hyperreaktivität, Hypervigilanz, kann schlecht stoppen)<br>· Vermindertes Verhalten (z.B. Depression oder fehlende Energie)<br>· Ausscheidungs-Problematik (z.B. Unsauberkeit im Haus bezüglich Urin und/oder Kot)<br>· Repetitive Verhaltensweisen, Stereotypien oder kompulsives Verhalten (z.B. Schwanzjagen, sich selbst übermässig belecken, im Kreis laufen, Schattenjagen)<br>· Reise-Probleme (z.B. kann sich im Auto nicht entspannen, bellen, Anzeichen von Reisekrankheit)<br>· Anderes

Umwelt

Wieviele Personen leben in demselben Haushalt wie Ihr Hund (einschliesslich Ihnen) ?	· Nur ich<br>· 2 Personen<br>· Mehr als 2 Personen
Bitte geben Sie an, wievielen Umwelreizen (z.B. Lärm, Verkehr, Personen, andere Hunde oder Tiere, ect.) Ihr Hund in der Wohnumgebung ausgesetzt ist/war.<br>· Für die jetzige Wohnumgebung<br>· Für die erste Wohnumgebung	Skala von 1-5, mit 1 = sehr wenig, 3 = mittelmässig, 5 = sehr viel<br>Ich weiss es nicht

Vorgeschichte Ihres Hundes

Aus welchem Land haben Sie ihren Hund ?	· Aus dem Inland<br>· Aus dem Ausland
Hatte Ihr Hund vor Ihnen noch andere Besitzer (ausser dem Züchter) ?	· Ja<br>· Nein<br>· Ich weiss es nicht

Persönlichkeit / Aktivität

Trifft eine oder mehrere der folgenden Merkmale auf Ihren Hund zu?	Antwortmöglichkeiten: ja / nein / ich weiss es nicht:<br><br>· ängstlich<br>· neurotisch<br>· neugierig<br>· sensibel<br>· schüchtern<br>· unsicher<br>· keines davon trifft zu
Wieviel Aktivität (Spaziergänge, Spielen drinnen oder draussen, Training, etc.) erhält Ihr Hund pro Tag ?	< 1 Stunde<br>1-3 Stunden<br>> 3 Stunden<br>Ich weiss es nicht
Wenn Ihr Hund sich einem neuen, unbekannten Objekt gegenüber findet, wie reagiert er/sie am ehsten?	· Er/sie hält inne und beobachtet aus der Distanz<br>· Er/sie fängt an zu bellen<br>· Er/sie geht dem Objekt aus dem Weg<br>· Er/sie bewegt sich auf das Objekt zu und beginnt damit zu spielen<br>· Keine der oben genannten Aussagen trifft zu<br>· Ich weiss es nicht

Frage-Nummer	**Der "Highly Sensitive Dog" Fragebogen**<br>Likert Skala von 1-7, mit 1 = trifft überhaupt nicht zu; 4 = trifft mehr oder weniger zu; 7 = trifft vollkommen zu<br>Option: Ich weiss es nicht
1	Mein Hund ist leicht gestresst, schnell mit Situationen überfordert.
2	Mein Hund bemerkt kleine Veränderungen.
3	Mein Hund ist schreckhaft.
4	Mein Hund wird schnell nervös oder ist häufig nervös.
5	Mein Hund scheint alles, was um ihn herum läuft, aufzusaugen.
6	Mein Hund reagiert darauf, wenn wir zuhause streiten.
7	Mein Hund ist eher unsicher und/oder vorsichtig.
8	Mein Hund ist ist emotional stabil, d.h. meistens ausgeglichen und nicht schnell aus der Fassung zu bringen.
9	Mein Hund ist tendenziell misstrauisch.
10	Mein Hund ist eher unruhig.
11	Mein Hund kann sich schnell an eine neue Umgebung gewöhnen und sich dort entspannen.
12	Mein Hund hat eine feine Wahrnehmung, d.h. er nimmt viel oder fast alles wahr.
13	Mein Hund hat Mühe mit Veränderungen im Tagesablauf (z.B. veränderte Routine, Besuch, etc.) und/oder mit Lebensveränderungen (z.B. Partnerwechsel, Umstellung von Möbeln, Ferien, Umzug, etc.).
14	Mein Hund reagiert schon auf geringe Veränderungen Intonation oder Lautstärke der Stimme.
15	Mein Hund braucht lange, bis er sich nach einer Erregung erholt und wieder "oben runter" kommt.
16	Mein Hund hat Mühe damit, wenn ihn Leute anfassen und/oder wenn ihn etwas berührt (z.B. Gstältli, Mantel, nasse Blätter, etc.).
17	Mein Hund ist reaktiv, d.h. er nimmt geringe Reize generell schnell wahr und reagiert schnell und/oder stark darauf.
18	Mein Hund ist aufmerksam.
19	Mein Hund scheint nachdenklich.
20	Mein Hund beobachtet alles, was um ihn herum geschieht.
21	Mein Hund hat "die Antennen immer ausgefahren".
22	Mein Hund ist generell entspannt, kann generell gut mit Stress umgehen.
23	Mein Hund hat Mühe damit, wenn ich ihn draussen warten lasse und mich ausser Sichtweite begebe.
24	Mein Hund reagiert stark auf Bestrafung.
25	Mein Hund ist fügsam.
26	Mein Hund ist sensibel.
27	Mein Hund ist fordernd.
28	Mein Hund ist emotional, d.h. er reagiert stark auf positive und / oder negative Ereignisse.
29	Mein Hund braucht Sicherheit.
30	Mein Hund ist leicht erregbar, "fährt schnell hoch", sei es durch positive oder negative Reize.
31	Mein Hund ist intelligent.
32	Mein Hund reagiert stark auf visuelle Reize.

Fragen, die für die Analyse umgekehrt werden müssen, d.h. 1=7, 2=6, 3=5, 4=4

Fragennummer	**Der "Highly Sensitive Person" Fragebogen**<br>Likert Skala von 1-7, mit 1 = trifft überhaupt nicht zu; 4 = trifft mehr oder weniger zu; 7 = trifft vollkommen zu
1	Sind Sie bei starken Sinneseinflüssen schnell überfordert?
2	Nehmen Sie Kleinigkeiten in Ihrer Umgebung wahr?
3	Beeinflusst Sie die Stimmung anderer Personen?
4	Neigen Sie zur Schmerzempfindlichkeit?
5	Haben Sie an Tagen, an denen viel läuft, das Bedürfnis, sich zurückzuziehen, sei dies ins Bett oder in ein dunkles Zimmer, oder an einen anderen Ort, an dem Sie alleine sein können und sich von der Stimulation erholen können?
6	Reagieren Sie besonders empfindlich auf die Wirkung von Koffein?
7	Sind Sie von Dingen wie hellen Lichtern, starken Gerüchen, grobem Stoff oder Sirenen in der Nähe schnell überwältigt?
8	Haben Sie ein erfülltes, komplexes Innenleben?
9	Bereiten Ihnen laute Geräusche Unbehagen?
10	Sind Sie durch Kunst oder Musik tief bewegt?
11	Fühlt sich Ihr Nervensystem manchmal so gespannt an, dass Sie alleine sein müssen?
12	Sind Sie gewissenhaft?
13	Erschrecken Sie leicht?
14	Sind Sie schnell gestresst, wenn Sie viele Dinge auf einmal in kurzer Zeit erledigen müssen?
15	Wenn sich Personen in einer Umgebung unwohl fühlen, wissen Sie im Allgemeinen, was getan werden muss, damit diese sich wohler fühlen (z.B. das Licht anpassen oder die Sitzordnung wechseln)?
16	Nerven Sie sich, wenn Leute versuchen, Sie dazu zu bringen, zu viele Dinge auf einmal zu tun?
17	Versuchen Sie immer zu vermeiden, Fehler zu machen oder Dinge zu vergessen?
18	Versuchen Sie aktiv, Filme oder Fernsehshows, die Gewalt beinhalten, zu vermeiden?
19	Fühlen Sie sich gestresst, wenn viel um Sie herum los ist?
20	Führt grosser Hunger bei Ihnen zu starken Reaktionen und unterbricht Ihre Konzentration oder Stimmung?
21	Rütteln Lebensveränderungen Sie auf?
22	Bemerken und geniessen Sie feine, zarte Gerüche, Geschmacksnuancen, Klänge oder Kunstwerke?
23	Ist es Ihnen unangenehm, wenn viel auf einmal läuft?
24	Hat es für Sie eine hohe Priorität, Ihr Leben so zu gestalten, dass aufrüttelnde oder überwältigende Situationen vermieden werden?
25	Stören Sie intensive Reize, wie laute Geräusche oder choatische Szenen?
26	Werden Sie, wenn sie mit anderen Menschen konkurrenzieren müssen oder bei einer Aufgabe beobachtet werden, so nervös oder unruhig, dass Sie viel schlechter abschneiden als sonst?
27	Haben Ihre Eltern oder Lehrer Sie als Kind als sensibel oder schüchtern betrachtet?
